# An Effective Translation: The Development of Hyaluronan-Based Medical Products From the Physicochemical, and Preclinical Aspects

**DOI:** 10.3389/fbioe.2018.00062

**Published:** 2018-05-17

**Authors:** Gloria Huerta-Ángeles, Kristina Nešporová, Gabriela Ambrožová, Lukas Kubala, Vladimir Velebný

**Affiliations:** ^1^Department of Research and Development, Contipro a.s., Dolní Dobrouč, Czechia; ^2^Free Radical Pathophysiology, Institute of Biophysics of the Czech Academy of Sciences, Brno, Czechia; ^3^International Clinical Research Center, St. Anne's University Hospital Brno, Brno, Czechia

**Keywords:** hyaluronan, chemical modification, cross-linked, hydrogel, preclinical data, clinical data, FDA, risk management

## Abstract

This review shows the steps toward material selection focalized on the design and development of medical devices based on hyaluronan (HA). The selection is based on chemical and mechanical properties, biocompatibility, sterilization, safety, and scale-up costs. These facts play a vital role in the industrialization process. Approved medical devices containing-HA are illustrated to identify key parameters. The first part of this work involves the steps toward a complete characterization of chemical and mechanical aspects, reproducibility of the processes and scale up. In a second stage, we aimed to describe the preclinical *in vitro* and *in vivo* assays and selected examples of clinical trials. Furthermore, it is important to keep in mind the regulatory affairs during the research and development (R&D) using standardization (ISO standards) to achieve the main goal, which is the functionality and safety of the final device. To keep reproducible experimental data to prepare an efficient master file for the device, based on quality and recorded manufacturing data, and a rigorous R&D process may help toward clinical translation. A strong debate is still going on because the denominated basic research in HA field does not pay attention to the purity and quality of the raw materials used during the development. So that, to achieve the next generation of devices is needed to overcome the limitations of state of art in terms of efficacy, biodegradability, and non-toxicity.

## Introduction

**Figure 1:**
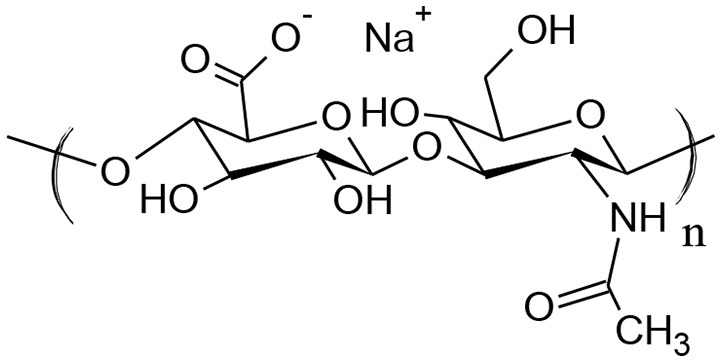
Structure of hyaluronan (HA).

Hyaluronic acid (HA), also referred to as hyaluronan is a linear polysaccharide ubiquitously present in the human body, which is found in the highest concentrations in synovial fluid, in eyes and skin. HA consists of alternating units of N-acetyl-β-D-glucosamine and β-D-glucuronic acid (Figure 1).

Traditionally, HA used for industrial applications was extracted from animal tissues i.e., umbilical cords or rooster combs (Shiedlin et al., 2004). HA isolated from living tissues is a mixture of several components, which does not satisfy the criteria of purity described by Pharmacopeia reference standard. Additionally, the purification and isolation procedures vary that it causes inconsistence between batches. Nowadays, bacterial fermentation is used for large-scale production. Besides, the production has been improved by metabolically-engineered recombinant bacteria strains that improved the yield (Kaur and Jayaraman, 2016).

The development of HA based-products is challenging because they are directly focused on human use. It is important to mention that the project manager based on an adequate risk assessment could mitigate many of the risks involved during the R&D (Table 1). HA-based products are covering a broad range of applications, which can be divided between cosmetic and therapeutic. The change of beauty standards led to increasing demand for cosmetics and asthetics procedures, including products made of HA. The dermal fillers containing-HA available in the market had to be approved by the Food and Drug Administration (FDA): Restylane®, Juvederm®, Bellotero®, Hylan B®, or Aliaxin®. In general, crosslinked HA is used as soft tissue fillers to eliminate wrinkles and shape the facial contour (Highley et al., 2016). However, the biological consequences of injecting these foreign materials into the dermis have not been studied to any great extent. Unfortunately, complications with these fillers can be difficult to address (Hwang, 2016).

**Table 1:** Risk assessment and evaluation toward designing a master file for a medical device product.

**Characteristics**	**Hazard (potential cause of failure)**	**Harm (potential event of failure)**	**Reduction measures**
**PREMARKET LIST**			
Raw materials do not meet desired specification	Unreproducible synthesis, purification, or isolation.	Unable to release/accept raw materials for processing into final device. Raised costs to manufacturer.	Implant an effective quality system. Control and monitoring of personnel and equipment.
Raw materials do not meet desired specification	Presence of impurities in the starting materials	Toxicity in the final product.	To characterize the raw materials.
The viscosity of the solution is low	Wrong choice of polymer	Incorrect function of the product.	To characterize the material following Pharmacopeia
The processing of the material is not reproducible	The polymer is not following specification.	Delays to production	To characterize the materials using highly sensitive methods.
Product is not possible to be sterilized.	Massive contamination during production or product handling. Degradation during sterilization.	Aseptic processing might not be feasible- product cannot be marketed	To study the effect of agents used for sterilization To change the way of sterilization.
**PRODUCT DESIGN**			
Mechanical properties of the product are not adequate for the application.	The material degraded during storage.	Incorrect function of the product.	Definition of mechanical properties according to state of art.
The material is not adequately stored.	Material decomposed	Incorrect function of the product	Characterization of the product before its use
The device is not stable.	Fast degradation.	Avoid of regeneration or efficacy.	To perform degradation studies *in vitro*.
***IN VITRO*** **USE**			
The material is cytotoxic.	Presence of impurities in the product.	Unable to be used *in vivo*.	To purify the product extensively. To develop validated and standardized *in vitro* assays of toxicity
The personnel do not correctly evaluate the material.	The cell model is not representative for the application	False biocompatibility.	Extensive *in vitro* characterization.
**ANIMAL MODEL**			
Macrophages are observed at the site of implantation.	Foreign body response is observed.	Limited biocompatibility.	Wrong choice of materials.
The material produces inflammatory responses in animals	Adverse reactions *in vivo*	Product cannot be translated	To develop an integrated strategy for toxicity testing *in vitro*.

Cosmetics products containing HA are increasing in the market and importance (Pavicic et al., 2011), as HA has been shown to be effective for the treatment of skin-aging (Papakonstantinou et al., 2012), skin, and wound repair (Tolg et al., 2014). For the above-mentioned reasons, HA is extensively used in many cosmetic preparations. As the market is relatively broad, many new preparations are currently under development; As an example, hydrophobized HA is able to encapsulate hydrophobic active compounds such as vitamins or antioxidants and acts as a carrier that helps the active compound to penetrate into dermis increasing its effect (Šmejkalová et al., 2017). Even though topical applications do not involve a very rigorous risk assessment, as they are externally used some regulations are followed. For controlling the use of prohibited substances on cosmetic products in Europe exists the regulation no. 1223/2009. Furthermore, as an effect of globalization, new markets in emerging countries are asking for strict quality controls to ensure the safety of cosmetic products. An urgent need for low cost, adequate, rapid methods is required, to allow the detection of forbidden compounds deliberately introduced in formulations. These methods should be able to identify of toxic components present as contaminants or impurities. Several proposals are emerging, and new regulations are expected.

In case of medical devices, the US and European regulatory agencies have established the classification to assure its safety and effectiveness. In Europe, there are four classes of devices ranging from low risk to high-risk (I, IIa, IIb, and III). Similarly, in the US there are three classes (I, II, III). The specific requirements and approval approaches for any medical device in EU and FDA were recently reviewed by (Van Norman, 2016). Most of the HA-based medical devices are classified as class III (in some cases as a class IIb in EU). For instance, HA is found in wound dressings, dermal fillers, anti-adhesive, osteoarthritis, ophthalmic, or vesicoureteral reflux devices. Between them, one of the most important medical applications is pain management (Migliore and Procopio, 2015) with eleven products receiving FDA approval for the treatment of knee osteoarthritis (OA) since 1997 (Doros et al., 2016). Intra-articular injections of HA, commonly referred to as viscosupplementation therapy have been classified in the US by the FDA as class III medical devices for more than 20 years. Two examples of these products are Monovisc®, a product currently in use in Europe (Laszlo et al., 2017) or Supartz®/Supartz FX™ for patients who failed to respond to non-pharmacologic therapy and simple analgesics in US market.

The first formulation approved by the FDA for use in the treatment of vesicoureteral reflux was stabilized HA/dextranomer (NASHA/Dx) (Geavlete et al., 2016). HA-based devices for the prevention of surgical post adhesion is also well-documented (Chen C.-H et al., 2017; Li et al., 2017); Seprafilm® is an anti-adhesion film to reduce abdominopelvic post-surgical adhesions, comprised of crosslinked HA with carboxymethylcellulose (CMC) (Diamond et al., 2012). This product was retired from the US-market due to allergic reactions in some patients.

HA is a natural lubricant and is well-suited for ophthalmic formulations (Battistini et al., 2017) and eye drops (Gross et al., 2017). Moreover, HA forms part of dietary formulations for the treatment of synovitis, knee pain, relief of synovial effusion or inflammation, and improvement of muscular knee strength (Oe et al., 2016).

This main goal of this review is to provide guidance to commonly used practices. Besides, this work intends to mention basic quality control parameters applied non-only to medical devices, but also to evaluate active ingredient based on ethics, and regulatory assessment and from a scientific and technological rationale. Table 2 enlists the three main norms applied for ensuring, both quality and safety of medical devices as discussed in this manuscript (Ibrahim and Chassapis, 2014; Bedi et al., 2017). However, the classification of medical devices and regulatory pathways are complex and vary from country to country (Vasconcelos et al., 2016).

**Table 2:** Evaluation of products requirements toward medical device with respective norms.

**Entry**	**Tasks in medical device development**	**Norms**
1	Chemical characterization of materials, degradation products, toxico-kinetics, sample preparation, sterilization, and residues. Preclinical studies (*in vitro* and *in vivo* testing)	ISO 10993
2	Risk assessment (identification of hazards, design and production, and clinical usage risks)	ISO 14971
3	Clinical safety, performance, and evidence	SG5/N2R8

## Physico-chemical characterization of native HA

### Design of HA specification under GMP practice

In the case of polymeric materials (such as HA) several parameters of characterization are requested. The procedures are described in Pharmacopeia and should follow the American Society for Testing Materials standards (ASTM standards) or ISO norms. Consequently, each product should be accompanied with a list of tests (specification) along with the analytical (validated) procedures used for the determination and appropriated acceptance criteria. The specification establishes the criteria to which a substance should conform to be considerable acceptable for its intended use. Apart from analytical tests and acceptance specifications, new chemical entities require the data from at least three independent batches to demonstrate the reproducibility of the manufacturing process.

For instance, a full characterization is carried following international standards. Norm ISO 14971 is a key standard for a manufacturer and identify the hazards associated with medical devices. This norm is used as a guide for evaluation of manufacturing processes, including up-scaling. All manufacturing processes should be performed under good manufacturing practice (GMP) and characterized by validated analytical methods. Norm ISO 14971 dictates that all the components of the device should be included in the analysis, including its source and purity. In the case of HA the presence of contaminants are reported to cause inflammatory responses even in extremely low concentrations (Šafránková et al., 2018). The analysis of raw materials and ingredients, as well as the quality of final products should be enunciated in the master file.

The required data for HA should be (i) description i.e. appearance, shape, color; (ii) identification/confirmation of structure (iii) assay describing the stability (iv) impurities describing the presence of organic volatile impurities and particulate matter, water content, residual chemicals, or solvents, degradation products. To address this, the final formulated sterilized product (medical device) is extracted (in both polar and non-polar solvents), and analyzed. The contaminants should be quantified by gas chromatography (GC-MS) as recommended by Pharmacopeia. Also, the specification includes physico-chemical properties, such as pH, viscosity, and particle size. Finally, microbial limits, endotoxins and pyrogens are enlisted. These contaminants (especially endotoxins and proteins) are not eliminated during sterilization and may be present in the finalized product (Baeva et al., 2017).

Native HA is used in medical devices as gel or solution. The absolute value of intrinsic viscosity identifies the material: i.e., HA with a value higher than 2.5 m^3^/kg, is recommended for parenteral administration and intra-ocular use, while a value of 0.3–0.6 m^3^/kg is recommended for cutaneous use; as an example, GenVisc 850® is a product containing highly pure HA (Doros et al., 2016), which is commonly used for the treatment of osteoarthritis knee pain. Unfortunately, the products found currently on the market differ drastically in rheological properties, and the relationship between viscosity and efficacy are still unclear (Wehling et al., 2017). Recently, more relevant techniques (tribological measurements) have been developed to elucidate the lubricating ability of HA formulations (Bonnevie et al., 2015). The tribology data effectively correlate the reduction of friction coefficient in cartilage with the viscosity and lubricant ability of a given formulation. Gigis et al. found that the efficacy might be related to the rheological properties and its origin (Gigis et al., 2016), but the existing data are still not possible to be correlated.

Nuclear magnetic resonance (NMR) is helpful for the chemical characterization of substances and mixtures in pharmaceutical formulations, medical devices, and drugs. Particularly, NMR identifies the structure of HA and impurities present in the samples. Therefore, this technique is applied to evaluate the downstream processes during the extraction and purification of HA and evaluates consistency between batches. A second technique is infrared spectroscopy (IR), a rapid procedure that provides a robust analysis and can supply functional-group information of samples.

### Structural elucidation of HA after chemical modification

Recently, chemically modified HA is the focus of active research for applications in biomedical device coatings, drug delivery systems, and scaffolds or cell-laden hydrogels for tissue engineering. Chemically modified HA is preferred due to the poor stability of native HA. It is important to mention that mild conditions for chemical modification are preferred due to possible degradation of HA during this process. Additionally, it is important to evaluate the cytotoxicity of the new formed chemical entities and its degradation products (Huerta-Angeles et al., 2016a). Additionally, reactive intermediates may change the native structure of HA or cause undesired cross-linking. So that, the new structure of HA is identified by a combination of analytic methods such as NMR, IR, and Mass Spectrometry (Picotti et al., 2013; Wende et al., 2016). However, chemically modified HA is not natural anymore, therefore, its biocompatibility cannot be assumed, even if the new chemical identity is based on natural components (Huerta-Angeles et al., 2017).

Chemical modification of HA can be roughly divided into two types based on the chemical or physical nature of the junctions: cross-linking and hydrophobization (Khunmanee et al., 2017). During the last decade, there are many works describing the covalently joint of several molecules and polymers to the HA-backbone. The most important parameter to evaluate after chemical modification of HA is the degree of substitution (DS) and the degree of cross-linking (DC). The DS of HA is commonly evaluated using ^1^H NMR or chromatographic techniques. DS is usually defined as an average number of side groups attached to 100 HA dimers. Therefore, the DS is expressed in % when DS equal to 10 % indicates that 10 out of 100 HA dimers are modified. DS can be determined by ^1^H NMR signals of HA anomeric H-atoms (from 4.3 to 4.6 ppm) or the methyl group located at 2.01 ppm corresponding to the -N-CO*CH3* in HA compared to the signals of the corresponding attached groups.

Dermal fillers are usually composed of cross-linked or stabilized HA, in a variety of chemistries and functionalities i.e., Hylaform® cross-linked with divinyl sulfone, or Aliaxin® with 1,4-butanediol diglycidyl ether (BDDE) (La Gatta et al., 2016). Particularly, a stabilized form of hyaluronic acid is necessary for aesthetic applications in order to increase the stability (Matarasso et al., 2006; Tran et al., 2014). In order to the determine DC in dermal fillers NMR is used (Guarise et al., 2012), carried out after enzymatic degradation (Wende et al., 2017).

In the case of hydrophobized HA, the use of ^1^H NMR is not always satisfactory for the determination of DS of high MW HA or for insoluble (or partially soluble) derivatives. In the last case, ^1^H NMR might overestimate the value due to more intense resonances of better soluble functional groups. Furthermore, the determination of the DS in hydrophobized HA can be performed after alkaline hydrolysis of the attached moieties, followed by its quantification by gas chromatography (GC), or Raman Spectroscopy (Chmelar et al., 2017). Similarly, Infrared spectroscopy (IR) reveals changes on the primary structure of HA after chemical modification. In the last case, a high degree of substitution is required due to the low sensitivity of the method (Choi et al., 2010; Huerta-Angeles et al., 2011).

Both DS or DC help to identify the process performance or product quality that is within the acceptable range, defined previously by customer standards. Also, it identifies defects in the process or the product itself.

### Molecular weight and polydispersity of HA

HA biological effects are unique among other biologically active molecules and are dependable on HA fragment size (Cyphert et al., 2015). Mw in specification is reported as average weight molecular weight (Mw) i.e., 7 × 10^5^ Da consists of polymer fragments of size from 3.4 × 10^5^ Da up to 1.4 × 10^6^ Da, while 16.9 kDa HA consists of oligosaccharides in the range of 2.2 × 10^3^–4.5 × 10^4^ Da. Therefore, it is important to include the value of polydispersity. Mw is a significant concern in the development of intra-articular injections of HA for the treatment of knee osteoarthritis (OA) due to striking differences in the product efficacy (Altman et al., 2016). Hylan G-F 20 (marketed as Synvisc®) is made of relatively high Mw (Mw of 6 × 10^6^ Da) (Migliore et al., 2010). High molecular weight HA is preferred for the last mentioned application (Bannuru et al., 2011; Zhao et al., 2016). Mw also determines the effectivity of systemic distribution and clearance of targeted organs; low molecular weight HA showed more rapid systemic distribution, while 6.7 × 10^4^ and 2.2 × 10^5^ Da showed longer persistence in the lungs (Kuehl et al., 2016). Mw changes the swelling degree and viscoelasticity of dermal fillers (Chun et al., 2016), while in cosmetic formulations is satisfactory the use of low molecular weight HA (2.0 × 10^4^–3.0 × 10^5^ Da) because it passes through the stratum corneum. In contrast, the impermeability of high molecular weight HA (1–1.4 × 10^6^ Da) through stratum corneum was reported (Essendoubi et al., 2016).

For all the above-mentioned reasons, it is important to identify the Mw of the polysaccharide (Braithwaite et al., 2016). A good method for the determination of the average molecular weight of native and modified HA is size exclusion chromatography combined with multiangle light scattering (SEC-MALLS) (Cožíková et al., 2017). Hydrodynamic characterization of HA and commercial products were characterized using SEC-TDA (Size Exclusion Chromatography-Triple Detector Array) (Salzillo et al., 2016).

### Sterilization of HA-based products

HA produces viscous solutions—particularly high molecular weight HA. The viscosity drastically decreased when the solutions are exposed to high temperatures (+100°C). Therefore, it can alter the properties of the medical device. However, HA-based products must be sterilized for their use.

Common sterilization methods include exposing HA-based materials to (i) ethylene oxide gas, (ii) dry or wet heat, (iii) electron beam, or (iv) γ-radiation. For liquids the preferred method of sterilization is filtration. The challenge is to sterilize the material with the minimal degradation (Liu et al., 2012). While all sterilization methods induce a decrease in the weight-average molecular weight (Drímalová et al., 2005), the irradiation methods, particularly γ rays, induced the highest decrease in Mw. Therefore, a post-sterilization validation (Mw determination) is essential to evaluate possible depolymerization of HA. UV absorbance measurement is also useful for the evaluation of possible degradation by an increase of absorbance due to the formation of double bonds due to oxidant conditions (Choi et al., 2010). In addition, rheology is used to evaluate the effect of sterilization heat cycles on the degradation of HA. Contaminants (especially endotoxins and proteins) are not eliminated during sterilization. In case of endotoxins, their concentration can even increase after the bacterial cell destruction during sterilization. The sterilization of medical devices is described under norm ISO 10993/7.

### Stability

The stability determination of HA-based products is crucial during a development (Olejnik et al., 2015). The stability studies should be carried out for new chemical entities as well as for finished products. The purpose of stability is to provide evidence on how the quality of any product varies as a function of time under several environmental factors i.e., temperature, humidity, and light. At selected time points, the characteristics of the materials are evaluated: structure, Mw, mechanical and thermal properties. A practical example, during the development of sodium linolenyl HA as a cosmetic ingredient (Huerta-Angeles et al., 2016b). A list of parameters was enlisted and evaluated for 12 months (Table 3) to ensure the product preserved its activity. Using stability data, a recommendation about storage conditions and shelf life are given. A second crucial point for a successful product outcome is the stability toward hyaluronidases action. (La Gatta et al., 2016). This parameter allows to predict the relative *in vivo* duration of the aesthetic effect of dermal fillers.

**Table 3:** Sodium linolenyl hyaluronate and its specification; the table provides the identity of each batch, acceptance criteria, and analytical methods used for the test (Huerta-Angeles et al., 2016b).

**Test**	**Specification limit value**	**Value**	**Method^a^**
Appearance	Visual	White or yellow granules	EP
Appearance of the solution (A_600_)	Clear	–	EP 2.2.2.5
Average molecular weight Mw_1_	10−30	kDa	EP method
Identification of sample (name, batch, structure)	Name and Number of batch	Pass	USP
Degree of substitution	7–13	%	NMR
Intrinsic viscosity	≥0.65 m^3^/Kg	Absolute value	EP 5.1472
Dry matter	>85	%	USP/EP
Loss of drying	<10	%	USP/EP
IPA	<0.5	%	Residual solvents, EP, pass
TEA	<0.03	%	Residual solvents, EP, pass
DMAP	<0.03	%	Residual solvents, EP, pass
Free linolenic acid (FLA)	<0.5	%	Residual chemicals, EP pass
Bacterial endotoxins	<100	CFU/g	EP 2.6.14
Heavy metals	<20	ppm	EP 2.4.8
pH of solution (0.5% in water)	5.0–8.5	–	EP 2.2.3

a *EP stands for European pharmacopeia and the number described the method of limits and analytical determination, USP stands for United states pharmacopeia*.

### Handling, packing, and storage

ISO 780 and ISO9001 norms are the standards to follow for handling and storage of HA-based products. A list of recommended actions is enlisted by the producer in the specification. Particularly, HA-based products are hygroscopic. Thus, the product should be stored in its original package in a clean and dry place, away from any sources of heat at low temperatures or room temperature (25°C). For HA-containing sensitive molecules, it is recommended that the product is stored at low temperature (4–8°C). Likewise, the products can be light sensitive, thereafter they are stored in dark or sealed package, or used immediately after opening. Unfortunately, it is not possible to describe a general rule as the stability varies between products, but it not expected to vary between batches. Finally, if the product is sterilized it should be used after opening and re-sterilization is not possible.

## Biological testing and safety assessment

HA-based products used as implants or with direct contact with damaged skin i.e., in wound healing, are classified as medical devices (Longinotti, 2014). To obtain FDA agency approval, the preclinical study submitted to the authority have to demonstrate that the intended product is safe. Preclinical information demonstrated that the product is not pyrogenic, mutagenic, toxigenic, hemolytic, or immunogenic. The guidance is included in norm ISO 10993. That norm also provides a framework for the biocompatibility evaluation based on three medical devices categories:

- Type of device (surface device, external communication device or implant)
- The duration of device/tissue contact (≤24 h, >24 h to 30 days, > 30 days)
- Type of tissue which is in contact with the device (e.g., intact skin, bone/tissue, blood etc.).

Cytotoxicity, sensitization, and irritation/intracutaneous reactivity assessment are recommended for all types of medical devices (Kim et al., 2014b).

HA is considered safe, but it possesses various biological functions depending on its molecular weight i.e., low Mw HA (oligosaccharides) are recognized by the immune system as damage-associated molecules patterns (DAMPs) and elicit inflammatory, angiogenic, and proliferative responses in various tissues. Mainly pro-inflammatory responses lead to foreign-body response (FBR) (Christo et al., 2015).

### HA-based materials *in vitro* cytotoxicity

The norm ISO 10993/5 governs the determination of cytotoxicity of medical devices, primarily the assessment of acute toxicity *in vitro*. Still, it leaves rather large freedom in choosing proper cell type and method of cell viability measurement. Many commercially available cell lines have been used: cultured fibroblasts from human skin, buccal mucosa, periodontal membrane or embryonic lung; the epithelial cancer cells (HeLa) as well as murine cells cultured from liver and spleen; T-lymphocytes from lymph nodes and macrophages (Wiegand and Hipler, 2009).

In the case of HA, the use of human keratinocytes (La Gatta et al., 2016), HaCaT (Sacco et al., 2016; Sun et al., 2017); murine cell lines [NIH-3T3 (Sigen et al., 2018), or L929 (Zamboni et al., 2017)] have been reported. It is advisable to employ cell type homologous with the tissue/organ concerns for the specific application, i.e., endothelial cells for testing stents (Choi et al., 2016; Hauser et al., 2017), human dermal fibroblasts and epidermal keratinocytes for wound healing (D'Agostino et al., 2015), adipose-derived stem cells for dermal fillers or gels (Guo et al., 2017; Stellavato et al., 2017) and chondrocytes for cartilage knee repair (Brittberg, 2014).

Based on the nature of tested medical device three types of cytotoxicity testing are proposed in ISO 10993/5: extract-dilution method, test by direct contact or indirect contact.

For most of the soluble (e.g., injectables) and some of the non-soluble medical devices, the extract-dilution test is the most common. In case of soluble compounds, the cells are cultured in media containing a range of concentrations of tested product. The semi-soluble and non-soluble devices (such as wound dressings containing HA) can be extracted into cultivation media and cells are then cultured with these extracts. Usually, the extraction is performed in full media (serum supplemented) for 24 h at 37°C. The presence of serum accelerates the extraction of leachables, solubilizes the substances and increases degradation, therefore, it affects the cytotoxicity of tested materials.

The extraction can be also performed without serum if it is more relevant for the intended application. It is important to mention that the extraction conditions should simulate as possible the conditions under which the device will be used. The extract-dilution method is more commonly adapted for the *in vitro* cytotoxicity evaluation of materials and devices directly used in the body. In addition, it is applied to a wide variety of raw materials and finished products that may release contaminants or toxic degradation products after continuous exposition.

The most often used read-out method to test cell growth rate and toxicity of the culture includes (i) metabolic assay such as neutral red uptake (NRU), MTT (methyl thiazolyl tetrazolium) T, XTT, resazurin assay, or ATP concentration measurement (ii) metabolism-independent method, i.e., crystal violet staining or DNA content measurement by PicoGreen. There are no reported interactions of HA with these methods. All these methods produce very similar results mainly in the determination of acute toxicity (≤24 h). According to the norm, these tests are performed only in a finished product. However, changes in the composition of the material can also influence the cytotoxicity tests. For instance, it is advisable to test the individual components in the composition during development. As an example, the presence of inorganic particles (e.g., in HA-based bone cement) can affect the spectroscopic assays. It is recommended to utilize methods with fluorescent or luminescent read-out (Kong et al., 2011) to evaluate effects of the individual components.

For water-insoluble HA-based products, the direct contact cytotoxicity test (or contact inhibition test) is used. The whole HA-based device or a piece is placed directly onto a cell monolayer and the subsequent changes of cell morphology and viability are assessed by microscopy, cell-staining, and viability measurement. This method is very sensitive but limited to specific devices i.e., very light or highly hydrophobic compounds will float and their attachment to cell monolayer is problematic. It is also necessary to determine if the HA-based device placement did not cause mechanical damage to the cell monolayer. This method enables even weak cytotoxicity to be detected because of its high sensitivity.

The indirect contact method includes molecular filtration and agar overlay test useful for the assay of leachables (Li et al., 2015).

A problem arises with slowly biodegradable devices such as cross-linked HA in fillers or anti-adhesives (Bhojani-Lynch, 2017). Some fillers are biodegradable in 12–18 months, or in 2–5 years (for slowly biodegradable fillers) as they are designed to last after implantation. Later, they are subsequently degraded and should be eliminated from the body without any cytotoxic reaction to both the intact material and its degradation products. Recent reports enunciated late inflammatory responses in patients (Wu et al., 2017). Prior to *in vitro* cytotoxicity testing, the biodegradable devices can be degraded in plasma or other suitable biological fluids with or without the addition of specific degrading enzymes, in particular, hyaluronidases for HA-based products (Li et al., 2015). Still, the translation from *in vitro* to *in vivo* conditions has limitations as the degradation rate depends on several factors such as the degree of modification of HA, impurities, manufacturing process, sterilization, device size, and the local tissue environment (hyaluronidase activity, inflammation) and the circulation rate (dilution of the degradation products). Similar difficulties were observed correlating *in vitro* and *in vivo* toxicity tests with other types of material, e.g., magnesium based implants, therefore, a modification of ISO 10993/5 cytotoxicity tests was proposed (Wang et al., 2015).

Nevertheless, a continuous progress is expected in the development of methods for cytotoxicity determination yielding more robust data with better correlation with *in vivo*. As an example, new methods include real-time measurement of cell attachment, and the real-time microscopic analysis including fluorescent viability assessment (calcein-AM assay, caspase activation assay etc.). However, these methods can only be used in standardized tests after proper optimization and validation. Finally, cytotoxicity tests are recommended for all medical devices as they allow a rapid evaluation, employ standard protocols, produce quantitative and comparable data, and due to their sensitivity, allow all the toxic materials to be withdrawn prior to animal testing.

### Other *in vitro* tests of HA-based materials

Apart from cytotoxicity, only sensitization and irritation tests are required for all types of medical devices. Nowadays, different models are currently validated to assess skin irritation *in vitro* i.e., the reconstructed human skin model (EpiDerm®, MatTek Corp.) (Hayden et al., 2015; Pedrosa et al., 2017).

The measurement of viability, histological analysis or molecular biology methods are determined in order to evaluate the complex response to medical devices (Casas et al., 2013; Coleman et al., 2015). Other *in vitro* tests include hemocompatibility (ISO 10993/4) and genotoxicity (ISO 10993/3). Hemocompatibility is recommended for devices that get in direct contact with blood. For example: cardiovascular devices (Turner et al., 2004), or acute wound dressings such as Hylan® (Longinotti, 2014), while genotoxicity assessment is recommended or required for implanted devices which are in contact with tissues for more than 24 h or more than 30 days, respectively: as an example, dermal fillers (De Boulle et al., 2013). Devices utilizing native HA are, in general, safe with no hemocompatibility (Simon-Walker et al., 2017), or genotoxic issues (Strand et al., 2012).

## *In vivo* tests of HA-based materials

The HA-based materials are recognized as being non-toxic in *in vitro* tests described above are subjected to *in vivo* tests. The norm ISO-10993/10 governs the biocompatibility testing of medical devices using guinea pigs, mice, and other small animals. The selection of particular *in vivo* test is dependent on the intended application of the product and vary significantly for materials intended for topical or internal applications. HA based-products intended to be used for internal application or application into open wounds (e.g., surgical or chronic wounds) are considered a medical device (Longinotti, 2014). These assays are governed by a broad range of national and international regulations and standards (e.g., CE mark or FDA 510(k) or PMA approval), but the regulatory requirements for medical devices are complex and vary between regions. In Europe, the regulations for active implantable medical devices (AIMD) is the EU Directive 90/385/EEC. The objective of the *in vivo* tests is to characterize the evolution of the tissue response after implantation of a medical device including its integration and the absorption/degradation.

The intended material for implantation should be manufactured, processed, cleaned of contaminants, and sterilized by the method intended for the final product. Subsequently, the samples to be implanted are aseptically handled to avoid contamination prior or during implantation. Surgery is performed under appropriate general anesthesia, carried out under aseptic conditions, and minimizing trauma. It is important to remove the hair from the surgical area and disinfecting the exposed area of skin. A small opening in the peritoneum is performed for the implantation and follows a well-established method for assessing the biological response and safety of any implant (Wortman et al., 1983). For example, this model was used for testing the effectivity of Seprafilm® (Morse et al., 2005).

An alternative method for assessing the biological response and safety of an implanted material is an implantation in the dorsal subcutaneous tissue of mice, rats, guinea-pigs, or rabbits (Pi et al., 2017). For example, the implantation of films made of HA tested by subcutaneous implantation into the backs of Wistar rats (Liu et al., 2005). An incision is made in the skin and one or more subcutaneous pockets are prepared by blunt dissection. In the last case, two or more pieces of material can be implanted but the gears should not touch each other.

The local effects of a new HA based-product are evaluated by comparison of the tissue response caused by the surgical procedure (sham controls). Furthermore, the effects of novel HA-based product are evaluated by direct pair comparison of two products. The first one, a reference (specimen) should refer to a product whose clinical acceptability and biocompatibility characteristics has already been established (Norm ISO 10993/6).

The tested-sample should be implanted into the tissues most relevant to the intended clinical use and evaluated in terms of mechanical and functional loading and safety. Normal rat and rabbit knee joints were used as a model to determine the tissue reaction to a material (Ishikawa et al., 2014); the inflamed or damage knee joint in rat or rabbit models was helpful for the evaluation of inflammation, joint lubrication, chondroprotective effects and antinociceptive effects (Gomis et al., 2009; Oliveira et al., 2014); air pouches established in BALB/c mice for the evaluation of inflammatory response of Hylan G-F 20 (Synvisc®) (Markel et al., 2014) and bilateral meniscectomies in sheep for assessment of the therapeutic potential of HYADD® (Smith et al., 2008).

Furthermore, it is important to consider the physical characteristics of tested sample (such as form, size, geometry, density, hardness, surface chemistry, swelling degree) because they influence the tissue response to the foreign material (Rayahin and Gemeinhart, 2017). In general, HA based-materials are degradable and absorbable with the consistence of a liquid or soft hydrogel (Khunmanee et al., 2017; Larrañeta et al., 2018). In this case, the swelling degree should be assessed as a considerable increased size of the implant is expected because HA binds a large amount of biological fluids. The determination of swelling helps to mitigate the risk of a potentially harmful situation of overdosing or mechanical damage.

The process of HA based-materials degradation and integration are key factors. Degradation studies *in vitro* should help to establish the proper time points for *in vivo* evaluation. Therefore, before starting preclinical studies with degradable materials, relevant information providing the degradation rate should be considered (Zhang et al., 2016), as tissue reaction on degradable materials is very different to non-degradable materials. The clinical exposure time to the device determines the test period. The preclinical study should span the degradation period for the device: (i) early period (when minimal degradation occurs) (ii) mid-period (when degradation is taking place) (iii) late period (when the implant is essentially absorbed). During the material degradation process, chronic inflammation can be also observed (Morais et al., 2010). Shortly after implantation, the reaction due to the surgical procedure itself is difficult to distinguish from the reaction caused by the implant. Finally, a homeostatic state of the tissue is expected after complete absorption of the material.

### Evaluation of *in vivo* testing

The health of the animals is observed and recorded minimally once per 24 h. After the animal has been humanely euthanized, the implant site is excised together with sufficient unaffected surrounding tissue to enable evaluation of the local macroscopic and histopathological responses (D'Este et al., 2016). The sample for histopathological analysis should include the tissue debris. The organs sensitive to systemic or local damage should be also collected (lymph nodes, liver, kidneys, spleen) for evaluation of subchronic systemic toxicity (Kim et al., 2014a).

Parameters of biological response to the tested material which should be assessed and recorded according to ISO-10993:

- Inflammatory and fibrotic response to the tested material;
- Degenerative changes in the tissue morphology;
- Number and distribution of the inflammatory cells (polymorphonuclear cells, lymphocytes, plasma cells, eosinophils, macrophages, and multinucleated cells) as a function of distance from the material/tissue interface;
- Signs of necrosis;
- other types of changes in tissues (e.g., vascularization, adipocytes infiltration, granuloma formation);
- Material characteristics (presence of fragments and/or debris, form, and location of debris);
- Tissue ingrowth to the material;
- Protocol (laboratory and personnel responsible for test, details of operations, description of test and control materials, description of animals, implantation and retrieval techniques and histological procedures, evaluation and observations of the above-mentioned parameters, statistical analysis) and final evaluation.

### Foreign body response to HA-based materials

Inflammatory reactions due to limited biocompatibility induces foreign body response (FBR). All materials implanted into living tissue initiate some host response as the first step of tissue repair (Franz et al., 2011). This process arises in consequence of the host reactions after implantation of the (bio)material and involves blood/plasma proteins adherence to the implant (acute inflammation). Moreover, monocytes/macrophages occurrence is observed at the site of implantation, together with foreign body cells formation, and extracellular matrix overexpression (chronic inflammation) (Anderson et al., 2008). Bruising, swelling, edema, infections, lumps and bumps, skin discoloration, and biofilm formation are common complications due to FBR (Urdiales-Gálvez et al., 2018). Even though, the risk associated to the use of HA is low, the use of cross-linkers may induce FBR. As an example, 0.6% of patients injected with Restylane® documented an incidence of hypersensitivity, divided equally between immediate and delayed reactions (Bitterman-Deutsch et al., 2015; Pérez-Pérez et al., 2017). There are reported cases with different characteristics i.e., a case of cellulitis-like FBR after HA-dermal filler injection (Shin et al., 2018). For the above mentioned reasons, FBR and the fate of scaffolds implanted should be cautiously evaluated in the animal model (Dondossola et al., 2016). Also, it is necessary to describe the method that will be used to extrapolate from the animal data to an effective regimen in humans. Safety studies obtained after preclinical findings allows the translation of a medical device into the clinics (Phillips and Wang, 2017).

## Clinical information

Extensive and complete documentation must be submitted to by the competent authority in the European Union, Japan, or the United States to obtain a marketing-authorization of a new HA-based therapeutic product. One of the most critical documents to be submitted is the clinical study report (CSR), which represents the integrated full report of efficacy and safety data. The only document available from a regulatory authority is the guideline issued by the FDA in 1999, which recommends the required information to be included (Alfaro et al., 2007).

In the case of dermal fillers, Hylaform® (Gold, 2007), Juvéderm® (Romagnoli and Belmontesi, 2008), Belotero Balance®, and Restylane® were approved by the FDA. All of them have demonstrated to be safe after being injected intradermally into the iliac crest region in 15 subjects (Tran et al., 2014). The clinical use of Seprafilm® was supported by preclinical and animal studies relating to surgical and obstetrical/gynecological applications (Diamond et al., 2012). HA was used also as a targeted transport vehicle of irinotecan in the treatment of metastatic colorectal cancer and underwent randomized Phase II clinical trial in 2005 (Gibbs et al., 2008). HA-based scaffolds (Hyalograft C) in the treatment of knee cartilage defects also reported preliminary clinical findings (Pavesio et al., 2003). The efficacy of Viscoderm® was demonstrated in patients undergoing facial rejuvenation procedures (Iannitti et al., 2016).

Randomized clinical studies have shown efficacy within 3 weekly intra-articular injections of Hylan G-F 20® (Boutefnouchet et al., 2017). Similarly, Hymovis® also underwent clinical studies and demonstrated effectivity and safety (Priano, 2017).

## HA products development toward new medical applications

In this section, we would like to address potential medical devices and medical device-related technologies that are likely to generate significant innovation over a ten-year period, based on reported cases (Kim et al., 2017). HA-based hydrogel was able to restore brain function following ischemic stroke (Nih et al., 2016). Using self-assembled HA to deliver agents to a cancer tumor has attracted increasing attention in the recent decade (Smejkalová et al., 2014; Chen M. et al., 2017; Quinones et al., 2018). The targeting properties and biodistribution of HA-based nanoparticles is very promising for atherosclerosis treatment (Beldman et al., 2017). Using HA hydrogels as tissue analog for stem cells-based therapies are merging as a promising strategy (Loebel et al., 2017; Wong et al., 2017). Oral administration of HA for the prevention of dry skin was established (Kawada et al., 2015; Kimura et al., 2016). The use of fragments of HA (35 kDa) was proposed as an additive in infant milk formula promoting intestinal defense (Kessler et al., 2018).

Still, the translation of these innovative approaches to clinics has been hindered by the poor correlation between *in vitro* and *in vivo* models. The same could be predicted for animal and human *in vivo* response.

## Overview of gaps, challenges, and potential opportunities in HA-based medical devices

More than 80 years after HA discovery we still lack the fundamental understanding of HA biological role. While biologists continue to unravel the complex biological functions of HA, the problem is complex, therefore, it some cases is not clear for the developer, whether a device works because HA is biologically active or because of its physical properties. HA cannot be considered a “filler,” because HA presents many biological functions and regulates immune responses. Therefore, a deeper understanding of the mechanisms underlying the roles of HA in various physiological processes can provide new insights and tools for the engineering of new medical devices.

Up to now, the role of HA in wound healing, chronic inflammation and cancer is not well-understood. Even now, new HA-binding proteins or HA-degrading enzymes are being described (Yamamoto et al., 2017) adding to HA's complexity. Moreover, the presence and biological effect of HA in healthy and diseased biological fluids and tissues is poorly understood (Cowman, 2017). The incidence of long-term adverse reactions secondary to the injection of a foreign material is still ignored. Thus, clinicians should be aware of the fate to these injectable agents. Improving the understanding of required steps toward the registration of a new a product is helpful for researchers. Increasing the knowledge of the therapeutic (and non-therapeutic) uses of HA through the understanding of clinical effects, safety, and efficacy is required.

Potential opportunities of the use of HA based materials and devices such as dry eye syndrome devices due to the aging of the population can be envisaged on the market. Additionally, the commercialization of oral HA-based formulations is expected. The global HA market was valued at USD 7.2 billion in 2016 and is growing over the forecast period.

## Conclusions

The natural origin of HA is both its benefit and its shortcoming in development of HA-based medical devices. In its native state, HA is highly biocompatible and biodegradable. HA is recognized by several cellular receptors and is degraded by enzymes which substrate specificity, expression profile and involvement in different pathophysiological processes vary. Moreover, it is still not clear how are various biological responses to HA directed by its structure and molecular weight. HA is a paradoxical molecule—is not the simple linear polymer because it forms part of very biological processes not well-understood. The chemical derivatization and processing into different forms and materials just add another level of complexity. Moreover, chemical modification broadens the spectrum of HA therapeutic applications.

Finally, it is impossible to separate biocompatibility and safety from performance and efficacy. Thus, the high-quality assessment of chemical, physical, and biological properties of HA-based products is mandatory. Collecting enough information of a well-characterized-product and an adequate risk assessment will provide an effective translation from the lab bench to the clinic.

## Author contributions

GH-Á revised the state of art of current devices based on HA and wrote the manuscript. KN evaluated all the necessary steps for *in vitro* characterization. GA and LK evaluated the data corresponding to animal model and *in vivo* testing. VV revised the manuscript.

### Conflict of interest statement

GH-Á and KN are currently employed by the company CONTIPRO a.s. VV is CONTIPRO's CEO. The other authors declare that the research was conducted in the absence of any commercial or financial relationships that could be construed as a potential conflict of interest.
